# Supplementary material for: Nek2A prevents centrosome clustering and induces cell death in cancer cells via KIF2C interaction
Source: Cell Death Dis. 2024 Mar 16;15(3):222. doi: 10.1038/s41419-024-06601-0 (PMC10944510; doi:10.1038/s41419-024-06601-0)
Supplement: Supplementary file 4 — un-cropped western blots [file 41419_2024_6601_MOESM4_ESM.pdf]

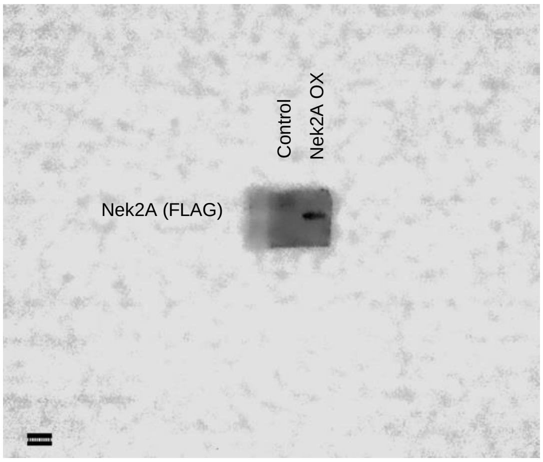

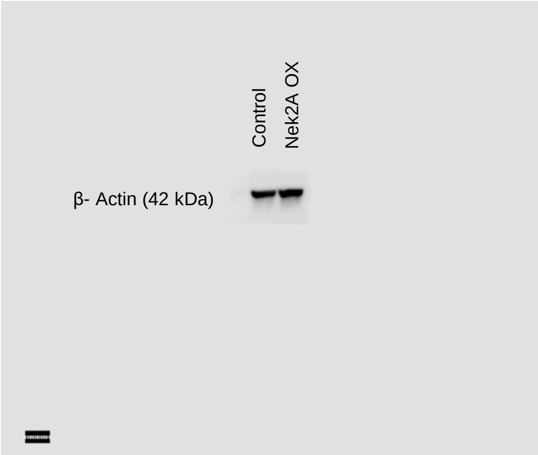

Western Blot images shown in **Figure 1B**

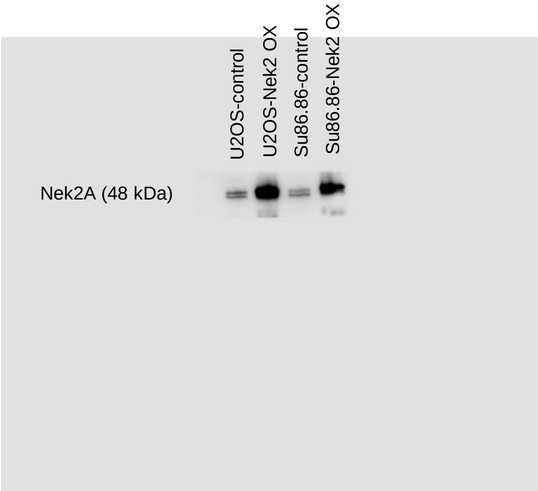

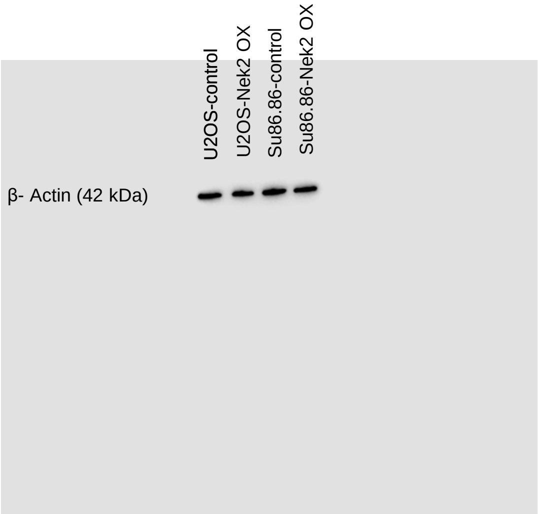

Western Blot images shown in **Figure 1C** (Su86.86 cells only) and **Supp. Fig. 1F** (U2OS cells only)

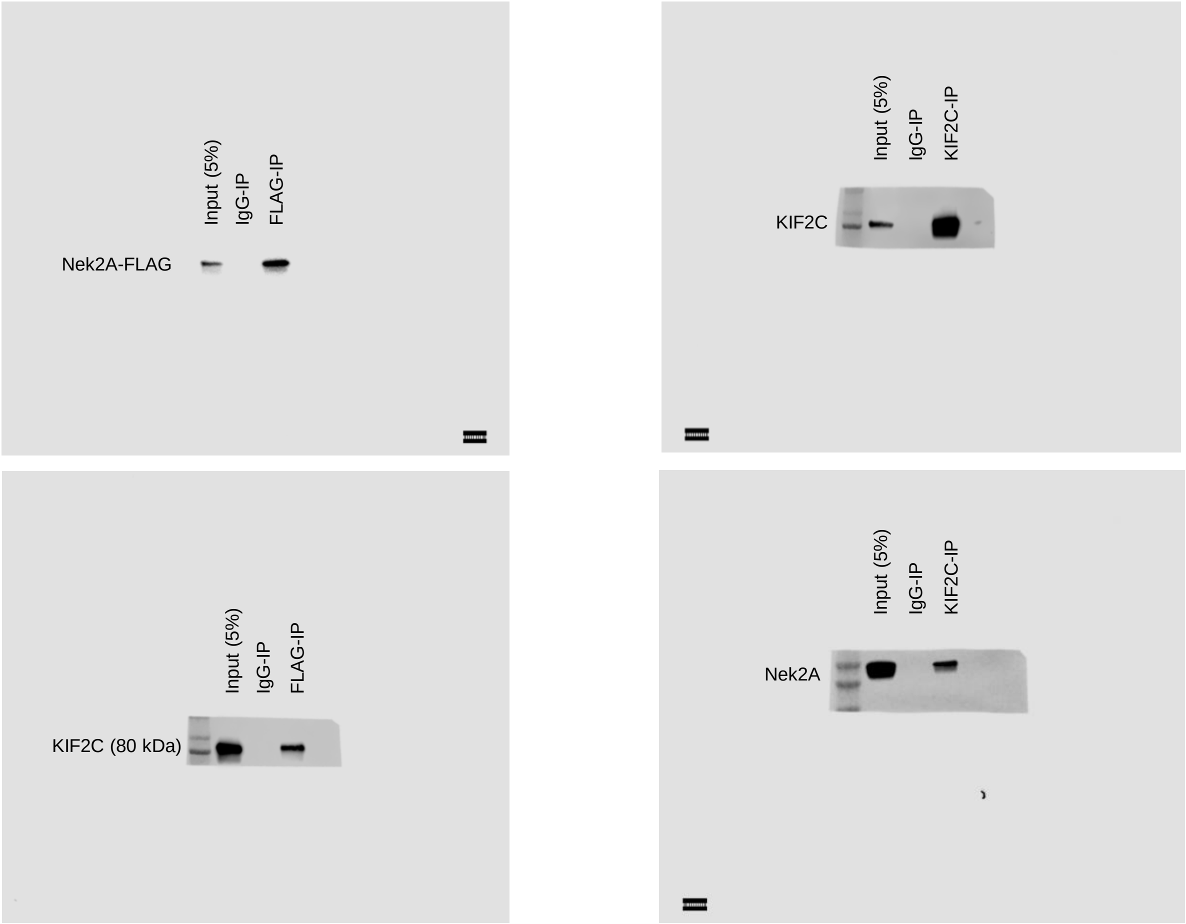

Western Blot images shown in **Figure 5H**

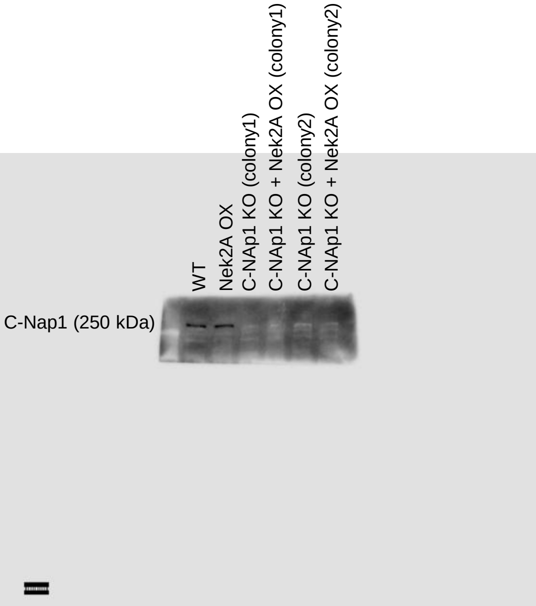

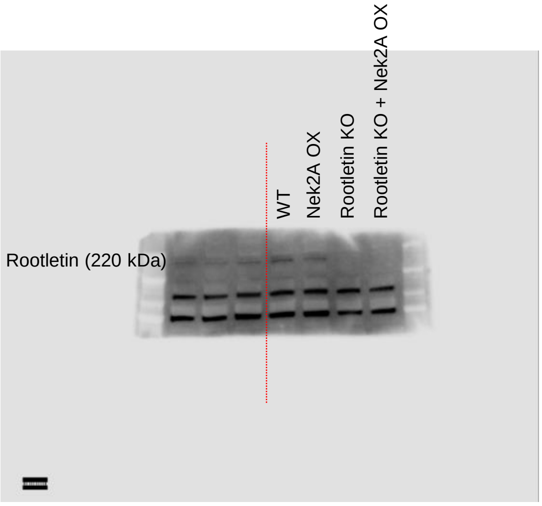

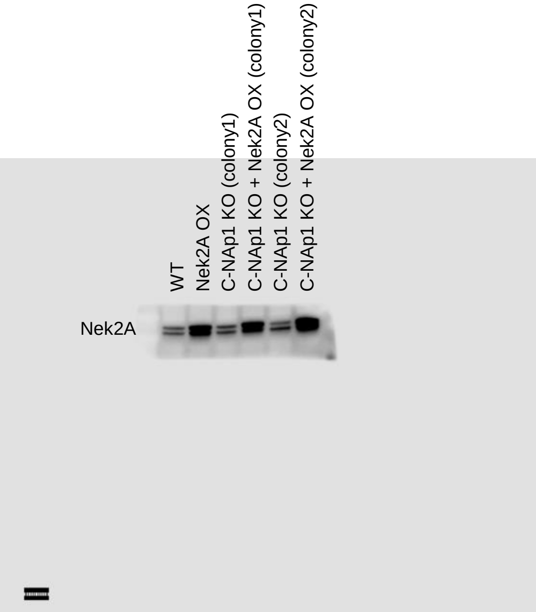

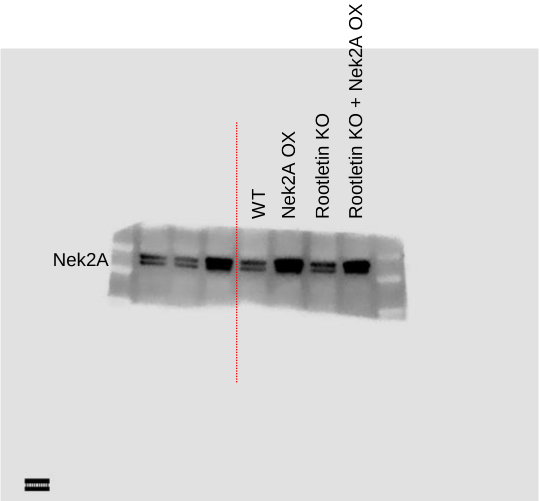

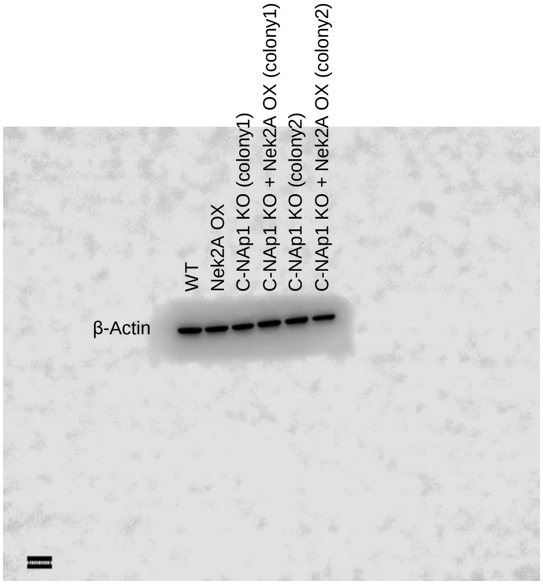

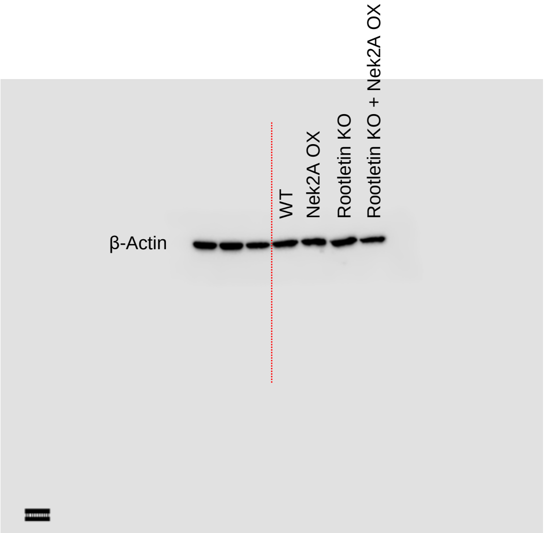

Western Blot images shown in **Supp Fig 4A-B**

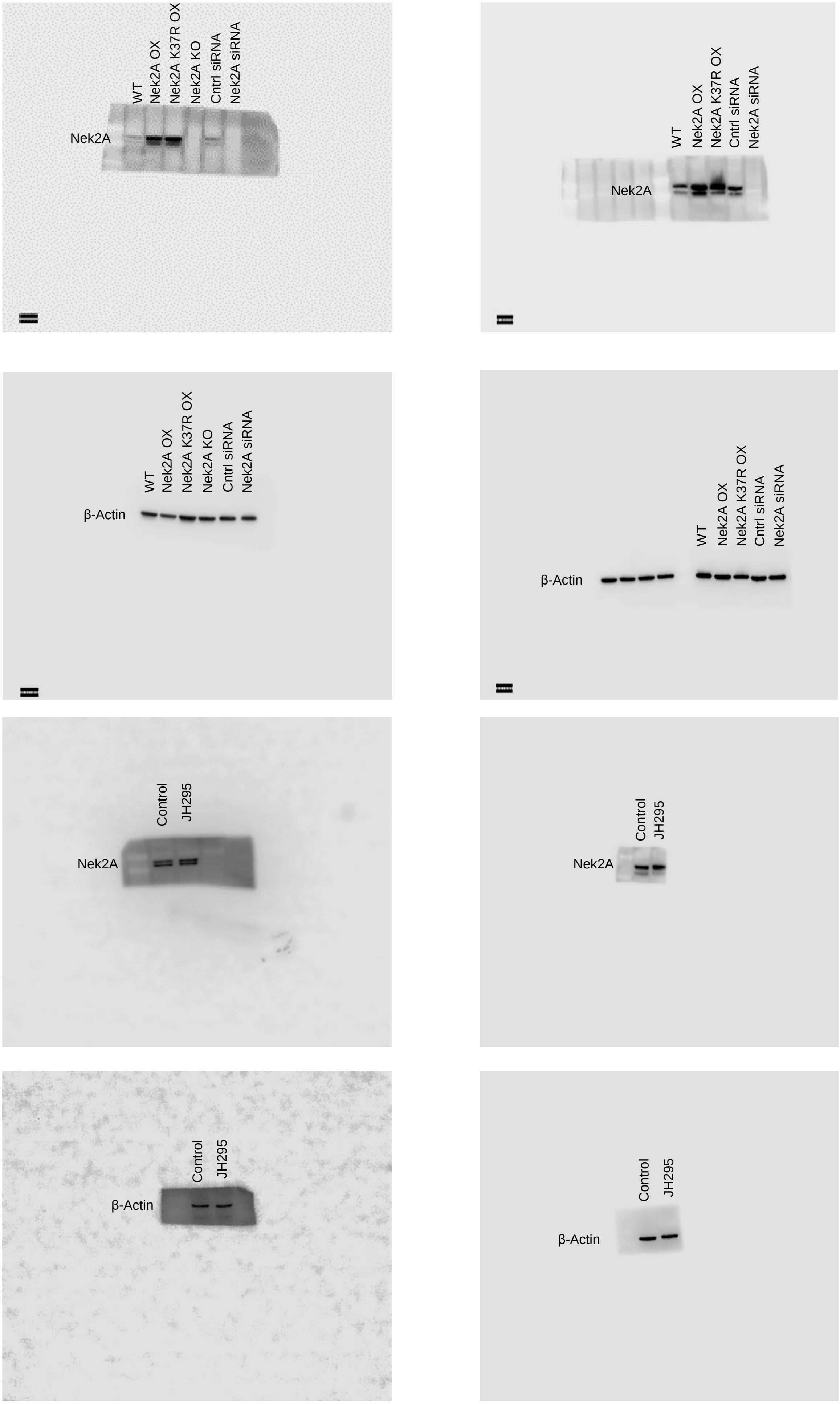

Western Blot images shown in **Supp Fig 2D**
